# Unraveling the distinctive gut microbiome of khulans (*Equus hemionus hemionus*) in comparison to their drinking water and closely related equids

**DOI:** 10.1038/s41598-025-87216-z

**Published:** 2025-01-22

**Authors:** Víctor Hugo Jarquín-Díaz, Anisha Dayaram, Eirini S. Soilemetzidou, Amelie Desvars-Larrive, Julia Bohner, Bayarbaatar Buuveibaatar, Petra Kaczensky, Chris Walzer, Alex D. Greenwood, Ulrike Löber

**Affiliations:** 1 Department of Wildlife Diseases, Leibniz Institute for Zoo and Wildlife Research, Alfred- Kowalke Str. 17, 10315 Berlin, Germany; 2 Experimental and Clinical Research Center, a cooperation between the Max-Delbrück-Center for Molecular Medicine in the Helmholtz Association and the Charité – Universitätsmedizin Berlin, Berlin, Germany; 3 Max-Delbrück-Center for Molecular Medicine in the Helmholtz Association (MDC), Berlin, Germany; 4 Charité – Universitätsmedizin Berlin, AG Rosenmund, Charitéplatz 1, 10117 Berlin, Germany; 5 Research Institute of Wildlife Ecology, University of Veterinary Medicine, Vienna, Austria; 6 Wildlife Conservation Society, Mongolia Program, Ulaanbaatar, Mongolia; 7 Department of Forestry and Wildlife Management, Inland Norway University of Applied Sciences, Stor-Elvdal, Norway; 8 Wildlife Conservation Society – Global USA and University of Veterinary Medicine AT, New York, USA; 9 School of Veterinary Medicine, Free University of Berlin, Oertzenweg 19 b, 14163 Berlin, Germany; 10 Present Address: Unit of Veterinary Public Health and Epidemiology, Complexity Science Hub, University of Veterinary Medicine, Vienna, Austria

**Keywords:** Microbiome, *Equus hemionus hemionus*, Freshwater microbiota, 16S rRNA full length gene sequencing, PacBio, Gobi Desert, Ecology, Microbiology, Zoology

## Abstract

The microbial composition of host-associated microbiomes is influenced by co-evolutionary interactions, host genetics, domestication, and the environment. This study investigates the contribution of environmental microbiota from freshwater bodies to the gastrointestinal microbiomes of wild khulans (*Equus hemionus hemionus*, *n* = 21) and compares them with those of captive khulans (*n* = 12) and other equids—Przewalski’s horse (*n* = 82) and domestic horse (*n* = 26). Using PacBio technology and the LotuS pipeline for 16S rRNA gene sequencing, we analyze microbial diversity and conduct differential abundance, alpha, and beta diversity analyses. Results indicate limited microbial sharing between wild khulans and their waterhole environments, suggesting minimal environmental influence on their gut microbiomes and low levels of water contamination by khulans. Wild khulans exhibit greater microbial diversity and richness compared to captive ones, likely due to adaptations to the harsh nutritional conditions of the Gobi desert. Conversely, captive khulans show reduced microbial diversity, potentially affected by dietary changes during captivity. These findings highlight the significant impact of environment and lifestyle on the gut microbiomes of equids.

**Supplementary Information:**

The online version contains supplementary material available at 10.1038/s41598-025-87216-z.

## Introduction

Organisms and the environments in which they live in sustain unique microbiomes. However, these are not entirely separated as animals shed exudate into the environment and are exposed to or consume microorganisms from it. In addition, the association between host and environmental microbiomes is dynamic. Over the last several millennia, human-animal interactions have changed with both the domestication of animals and the keeping of captive wildlife, resulting in changes to the associated microbiomes^1^. Yet, it is still being determined how strong the interdependence is between animals, microbes, and their environment.

Crucial indicators of ecosystem health are the microbiomes in freshwater and sediment^2^ and water quality assessment including fecal bacterial contamination. The diversity of animal species in these environments enhances the microbial richness and complexity of the overall ecosystem. Additionally, the various animal species harbor unique microbial communities within their bodies, further contributing to the environment’s ecological balance and overall bacterial composition. Understanding host-environment microbiome dynamics provides insights into bacterial community sharing, including potential pathogens^3^. Waterholes have been identified as viral vectors for free-living equids, for example, equid herpesviruses (EHVs) in sediment and water samples. The in-vitro infectivity of EHVs was confirmed through the successful isolation of viral particles and further cell culturing of EHV-1 from Mongolian water samples^4^. Additionally, an association between water scarcity and increased EHV shedding in zebras suggests a physiological stress response of the hosts, while potential viral transmission through water may increase transmission risk in such contexts. These interactions between viruses and their equid hosts at waterholes underscore a complex web of ecological relationships, which are part of broader host-microbiome coevolutionary processes that have unfolded over millions of years. Thus, changes in the abundance of prevalent microbial species may have major consequences for the health of the host^5^. In captivity, the microbiomes of both domesticated and wild animals are likely to have undergone major shifts due to dietary changes, loss of habitat, reduced social interactions and use of antibiotics^6,7^. Such a shift was documented in the fecal microbiomes of the Przewalski’s horse (*Equus ferus przewalskii*). Przewalski’s horses raised in captivity had much lower fecal microbiota diversity than individuals born in the wild in Seer, Mongolia. Furthermore, Przewalski’s horses in Mongolia had a higher abundance of specific plant taxa in their diet compared to domesticated horses in the same region^7^. The differences in gut microbiota diversity and composition between wild and domesticated horses may be influenced by diet, ecological habitat and grazing preferences. The results suggest that domestication may have led to a loss of gut microbiome diversity in horses, similar to what has been observed in humans transitioning to agricultural and urban lifestyles.

In this study, we employ full-length PacBio 16S rRNA gene sequencing to explore the potential contributions of water and sediment microbiota from waterholes in two distinct parts of the Mongolian Gobi Desert to the gastrointestinal microbiomes of wild khulans (*Equus hemionus hemionus*). By analyzing rectal swabs as proxies for their gastrointestinal microbiomes, we compare the microbial profiles of wild khulans with those of their captive counterparts residing in Serengeti Park, Germany. We hypothesize that the microbial diversity in the environment, particularly within drinking water, shares significant overlap with that found in the khulans, suggesting a shared microbial pool^7^. Additionally, we posit that captivity leads to a reduction in microbial diversity, reflecting the impacts of changed living conditions and diet. This study also extends to examining overlaps with previously published fecal microbiomes of Przewalski’s horses from Mongolia, providing insights into how environmental and lifestyle changes affect equid microbiomes^8^. Our use of PacBio sequencing not only tackles the challenges associated with the variability of 16S rRNA gene microbiomes but also enhances our taxonomic annotation at the species level, ensuring precise genetic identification and comparison.

## Results

### General sequence processing statistics

Of the 1,228,137 reads generated, 1,065,368 (min. 644 - max. 40,117 reads per sample) pass the pre-processing and quality check and are assigned to OTUs with the Lotus pipeline (Table S1). After OTU clustering, we implemented the following filtering of the data: (1) samples and OTUs with zero counts were discarded; (2) uncharacterized OTUs at Kingdom or Phylum level were omitted; and (3) we maintained those OTUs with total abundance higher than five reads and mean prevalence higher than one sample. The final dataset used for further analysis had 980,130 reads, 431,277 from khulan and 548,853 from waterhole samples, with a mean of 10,249 reads/sample and 9,616 rarefied and curated OTUs with an average of 261 OTUs/sample. Wild khulan samples had 2,735 (of 3,496) exclusive OTUs, while captive khulans accounted for 604 exclusive OTUs out of 1,334. Waterhole-derived samples had 2,137 out of 3,112 and 2,427 out of 3,420 exclusive OTUs for water and sediment, respectively (Figure S1).

### Wild khulan microbiomes are richer and more diverse than those of captive khulans and waterholes in the Gobi

**Fig. 1:**
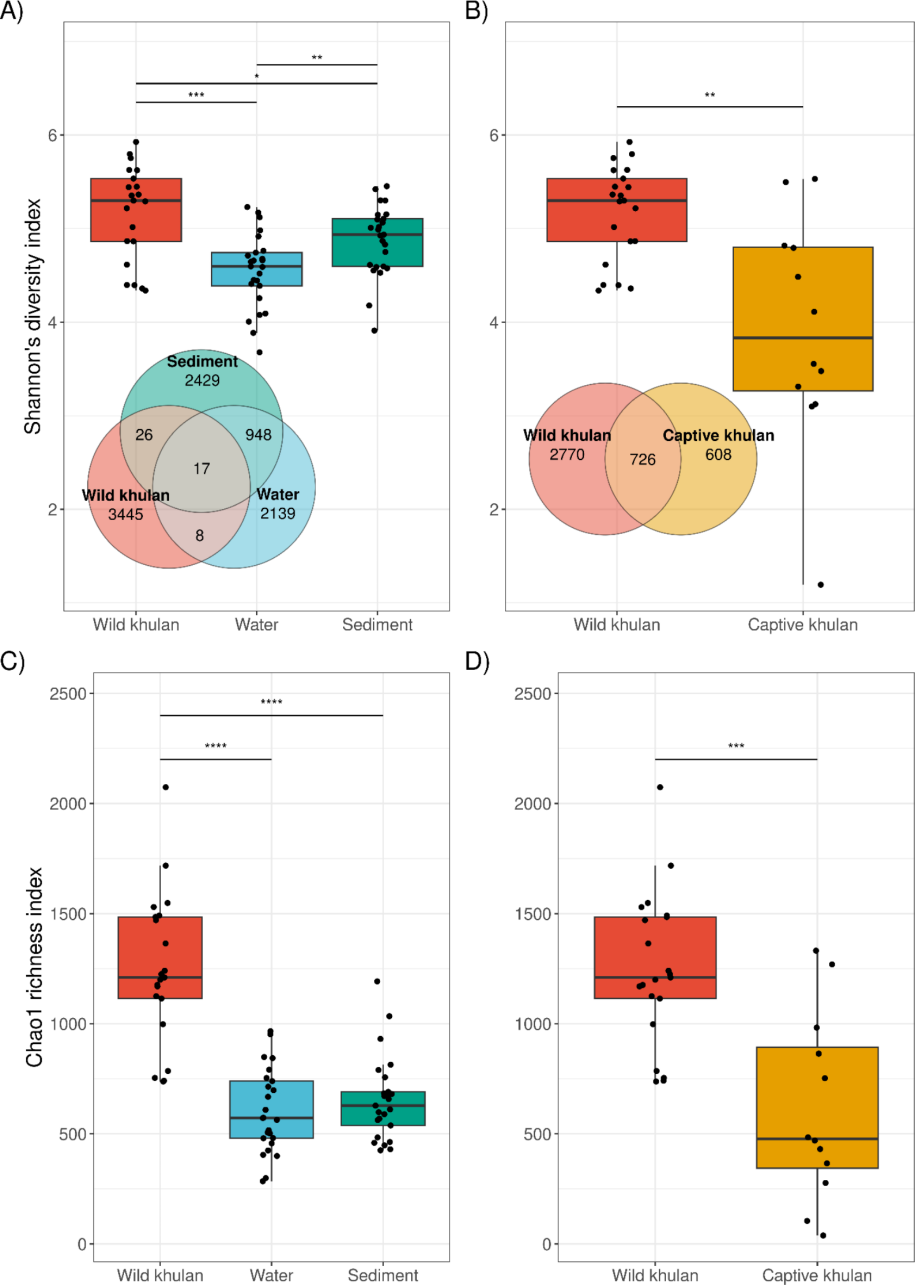
Alpha diversity metrics of the microbiome of wild khulan, captive khulan and waterholes in the Gobi. Wild khulan microbiomes are significantly more diverse and rich than waterholes (**A**,**C**) and captive khulans (**B**,**D**). Wild khulans shared 17 OTUs with environmental samples from waterholes and 726 OTUs with captive khulan.

Alpha diversity metrics were compared to determine how different wild khulan microbiomes are compared to those from (1) waterhole samples taken where they congregate in the Gobi and (2) Captive khulan. Shannon’s index comparisons indicates that wild khulan microbiomes are significantly more diverse than both types of environmental samples, water (Mann-Whitney *U*,* U*(*n*_wild_= 21, *n*_water_= 25) = 425, *Z* = 3.584, *r =* 0.528, *p*_*FDR.adjs*_ = 0.0006) and sediment taken from the same location (Mann-Whitney *U*,* U*(*n*_wild_= 21, *n*_sediment_= 25) = 358, *Z* = 2.106, *r =* 0.311, *p* = 0.035) (Fig. 1A). A comparison of the Chao1 index also revealed a significantly higher richness of wild khulan microbiomes than waterhole samples (Fig. 1C). Wild khulan, water, and sediment microbiomes shared 17 OTUs and four of them with annotation at the species level: *Caenimonas terrae*, *Comamonas kerstersii* and *Pelomonas saccharophila* from the family Comamonadaceae, and *Ralstonia insidiosa* from the family Burkholderiacea, all of them widely distributed in environmental samples like water or soil^9,10^. In contrast, captive khulans shared only eight OTUs with environmental samples from Gobi (Figure S2).

Wild khulan microbiomes were more diverse (Mann-Whitney *U*,* U*(*n*_wild_= 21, *n*_captive_= 12) = 208, *Z* = 3.069, *r =* 0.534, *p*_*FDR.adjs*_ = 0.0015) (Fig. 1B) and richer (Mann-Whitney *U*,* U*(*n*_wild_= 21, *n*_captive_= 12) = 216, *Z* = 3.368, *r =* 0.586, *p*_*FDR.adjs*_ = 0.0004) (Fig. 1D) when compared to those from captive animals. Wild khulan microbiomes shared 726 OTUs with captive khulan microbiomes. Among these shared OTUs, Firmicutes from the families Lachnospiraceae (5.44%), Streptococcaceae (2.91%), and Oscillospiraceae (2.79%), together with families Bacteroidaceae (3.76%) and Commamonadaceae (2.5%) were the most abundant among khulan microbiomes. Out of the 19 OTUs with annotation at the species level, five had a prevalence of at least 20%: *Comamonas kerstersii* (20%), *Escherichia coli* (27%), *Flavonifractor plautii* (25%), *Pelomonas saccharophila* (31%), and *Ralstonia insidiosa* (22%). *C. kerstersii* had the highest relative abundance among khulans, with 1.82%. An uncharacterized *Streptococcus* sp. isolate 27284-01 was among those OTUs with high prevalence (26%) and relative abundance (1.51%) in khulans. While *C. kerstersii*, *P. saccharophila* and *R. insidiosa* are environmental bacteria, *E. coli*, *F. plautii* and *Streptococcus* isolate 27284-01 are known gut bacteria. The differences in read counts per sample did not significantly impact the alpha diversity measures (Figure S3).

### Microbial composition in wild khulans is more similar to captive khulans than to Mongolian waterholes

**Fig. 2:**
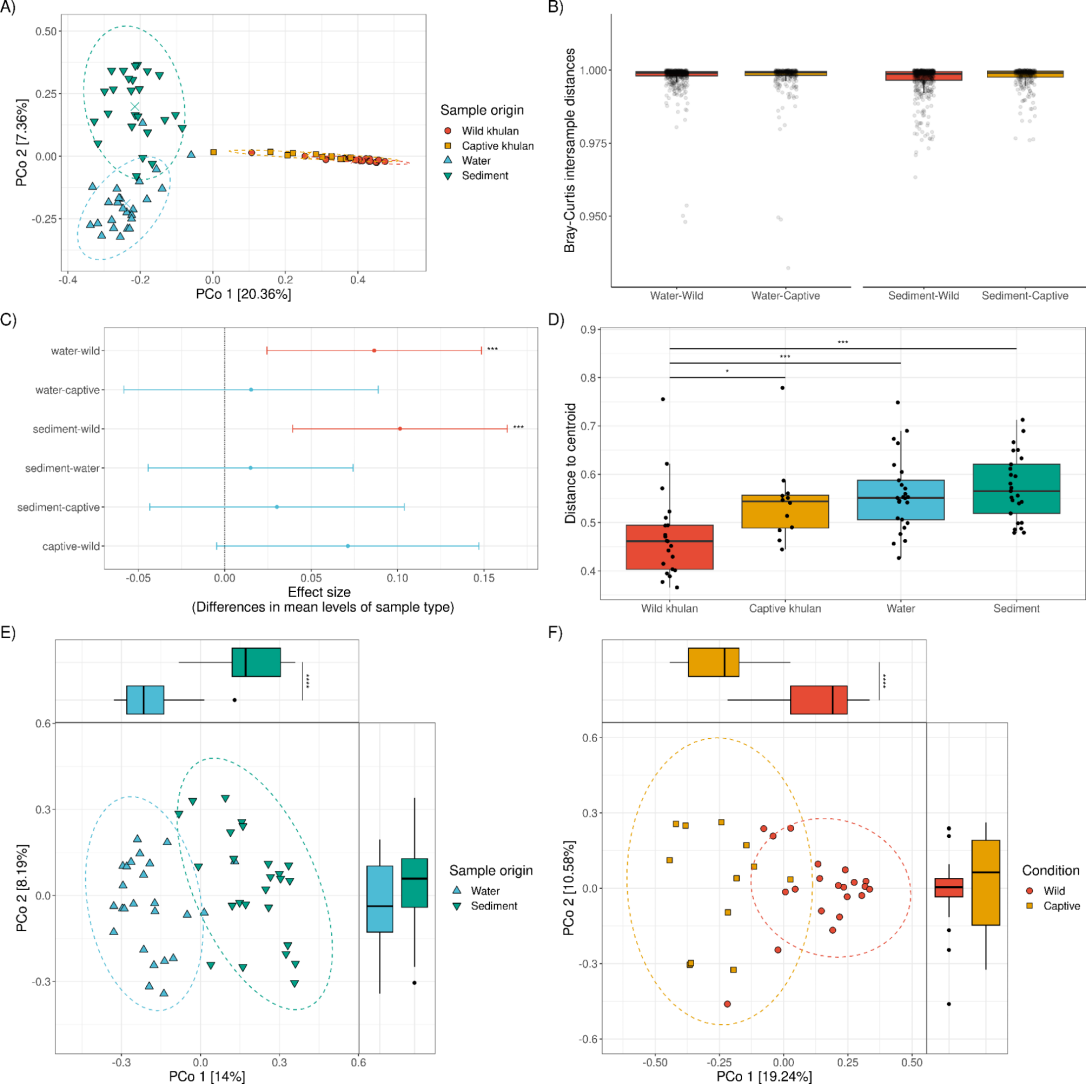
Beta diversity analysis shows distinct microbial communities in wild khulans, waterholes, and captive khulans. (**A**) Principal Coordinates Analysis (PCoA) plots comparing the microbial beta diversity of wild and captive khulans, water and sediments from waterholes. (**B**) Pairwise comparison of intersample Bray-Curtis (BC) dissimilarity distances shows no difference between wild and captive khulan to water or sediment microbial communities. Every dot represents the distance between a pair of samples. (**C**) Mean and the 95% confidence interval of a post-hoc Tukey’s HSD test performed on BC dissimilarity distances shows highly significant differences between wild khulan microbiomes, water and sediment microbiomes. (**D**) The overall comparison of multivariate homogeneity of sample type dispersions for BC dissimilarity distances between samples relative to the centroid shows a higher significant difference between wild khulan microbiomes to waterhole-derived samples. (**E**) PCoA from waterhole-derived samples confirms that 14% of the variation in PCoA 1 and the observed clustering in microbial composition varies significantly based on the sample origin. (**F**) Clustering of khulan microbiomes in the PCoA significantly depend on living condition, either wild or captive. Significance codes: ‘****’ 0.0001, ‘***’ 0.001 ‘**’ 0.01 ‘*’ 0.05.

Principal coordinate analysis (PcoA) based on the Bray–Curtis dissimilarity (BC) distance was used to determine how similar the bacterial composition of khulans and waterholes are. PCoA showed three clusters, representing the two waterhole-derived samples, water and sediment, respectively, and an additional cluster with overlapping wild and captive khulan samples (Fig. 2A). PERMANOVA indicates that the sample type, water, sediment, wild khulan or captive khulan, is a significant predictor of differences in microbial composition, explaining around 29% of the total variation (Table 1).

**Table 1:** PERMANOVA based on the Bray-Curtis distances between wild khulan and waterhole samples.

	Df	Sums Of Sqs	F-Model	*R* ^2^	*p*
Sample type	3	10.019	10.673	**0.2884**	0.001***
Residuals	79	24.721	-	0.7116	-
Total	82	34.741	-	1.0000	-

---.

Significance codes: ‘***’ 0.001, ‘**’ 0.01, ‘*’ 0.05, ‘.’ 0.1, Df: Degrees of freedom, *F*-Model: *pseudo F*-test statistic, R^2^: Variance explained and corresponding *p* value based on 999 permutations.

GLMMs with the living condition as a predictor of microbiome dissimilarity were used to ascertain the similarity between khulan’s microbiomes, either wild or captive, to waterhole microbiomes. The analysis revealed no significant difference between the microbiomes of the water (LRT: *χ*^2^ = 0.587, df = 1, *p* = 0.443) or sediment (LRT: *χ*^2^ = 1.934, df = 1, *p* = 0.164) samples and the wild or captive khulans. All animal-derived microbiomes were different in composition from waterholes; this is reflected by the BC distance above 0.9 and closer to 1.0 for all pairwise comparisons (Fig. 2B). Additionally, we confirmed that the geographical distance between wild khulans and the sampled waterholes had no significant impact on the the microbial composition of the water (Mantel, *R* = 0.112, *p* = 0.183) or sediment samples (Mantel, *R*= -0.151, *p* = 0.866), no matter if a higher or lower potential of visit to the waterhole is considered (Figure S5).

An analysis of variance on BC distances confirmed PERMANOVA results on significant variation among sample types (ANOVA, *F*_(3, 79)_ = 6.982, *p* = 0.0003). Further multiple pairwise comparisons of mean BC differences between sample types showed a clear significant difference between wild khulan microbiomes and waterhole samples (Tukey HSD, Mean diff_wild−Water_= 0.086, 0.024–0.149 95% CI, *p.adj* = 0.003; Mean diff_wild−Sediment_= 0.101, 0.039–0.164 95% CI, *p.adj* = 0.0003). A lack of significant difference between wild and captive khulan microbiome composition could reflect the closeness of both microbiomes (Mean diff_wild−Captive_= 0.071, -0.005–0.147 95% CI, *p.adj* = 0.074) (Fig. 2C). Nevertheless, the overall comparison of multivariate homogeneity dispersions for BC distances between samples relative to the centroid of the sample type clusters in the PCoA confirmed a significant difference between wild khulan microbiomes to waterhole-derived samples (Mann-Whitney *U*,* U*(*n*_wild_= 21, *n*_water_= 25) = 103, *Z* = 3.517, *r =* 0.519, *p*_*FDR.adjs*_ = 0.0008; *U*(*n*_wild_= 21, *n*_Sediment_= 25) = 78, *Z* = 4.069, *r =* 0.600, *p*_*FDR.adjs*_ = 0.0001). It also reflect more differences than expected between wild and captive khulan microbiomes (Mann-Whitney *U*,* U*(*n*_wild_= 21, *n*_Captive_= 12) = 56, *Z* = 2.620, *r =* 0.456, *p*_*FDR.adjs*_ = 0.016) (Fig. 2D). To further characterize the differences between microbiomes, waterhole derived microbiomes and khulan derived microbiomes were analyzed separately.

PCoA based on BC distances between water and sediment confirmed the overall difference in composition between sample types. PCo1 was explained mainly by the water and sediment differentiation (Mann-Whitney *U*,* U*(*n*_Water_= 25, *n*_Sediment_= 25) = 12, *Z* = 5.830, *r =* 0.825, *p* = 4.3E^− 12^), while for PCo2 it was possible to detect a significant impact of the geographical location from the waterholes (Mann-Whitney *U*,* U*(*n*_Gobi A_= 23, *n*_Gobi B_= 27) = 193, *Z* = 2.284, *r =* 0.323, *p* = 0.022). Neither the geographical location nor physicochemical characteristics at sampling time (the temperature or pH) significantly predicted waterhole microbial composition (Fig. 2E; Table 2).

**Table 2:** PERMANOVA based on the Bray-Curtis distances between water and sediment samples.

	Df	Sums Of Sqs	F-Model	*R* ^2^	*p*
pH	1	0.360	1.076	0.0197	0.32
Temperature	1	0.273	0.815	0.0149	0.801
Region	1	0.406	1.212	0.0221	0.151
Sample type	1	2.055	6.140	**0.1122**	0.001***
Residuals	45	15.063	-	0.8222	-
Total	49	18.321	-	1.0000	-

---.

Significance codes: ‘***’ 0.001, ‘**’ 0.01, ‘*’ 0.05, ‘.’ 0.1, Df: Degrees of freedom, *F*-Model: *pseudo F*-test statistic, R^2^: Variance explained and corresponding *p* value based on 999 permutations.

Despite the apparent substantial overlap in microbial composition between wild and captive khulan microbiomes (Fig. 2A), these khulan clustered separately based on the living condition of the animals (Fig. 2F) when analyzed without the waterhole samples. Wild versus captive significantly explained 14% of the variation in the data (Table 3). For PCo1, the living condition explained the clustering to a large degree (Mann-Whitney *U*,* U*(*n*_wild_= 21, *n*_Captive_= 12) = 242, *Z* = 4.34, *r =* 0.756, *p* = 7.8E^− 7^). PERMANOVA indicated that sex was no significant driver of the beta diversity (Table 3).

**Table 3:** PERMANOVA based on the Bray-Curtis distances between wild and captive khulans.

	Df	Sums Of Sqs	F-Model	*R* ^2^	*p*
Sex	1	0.267	0.978	0.027	0.414
Living condition	1	1.377	5.044	**0.140**	0.001***
Residuals	30	8.193	-	0.830	-
Total	32	9.870	-	1.0000	-

---.

Significance codes: ‘***’ 0.001, ‘**’ 0.01, ‘*’ 0.05, ‘.’ 0.1, Df: Degrees of freedom, *F*-Model: *pseudo F*-test statistic, R^2^: Variance explained and corresponding *p* value based on 999 permutations.

### Neither khulan nor waterhole microbiomes are enriched with potentially pathogenic bacteria

**Fig. 3:**
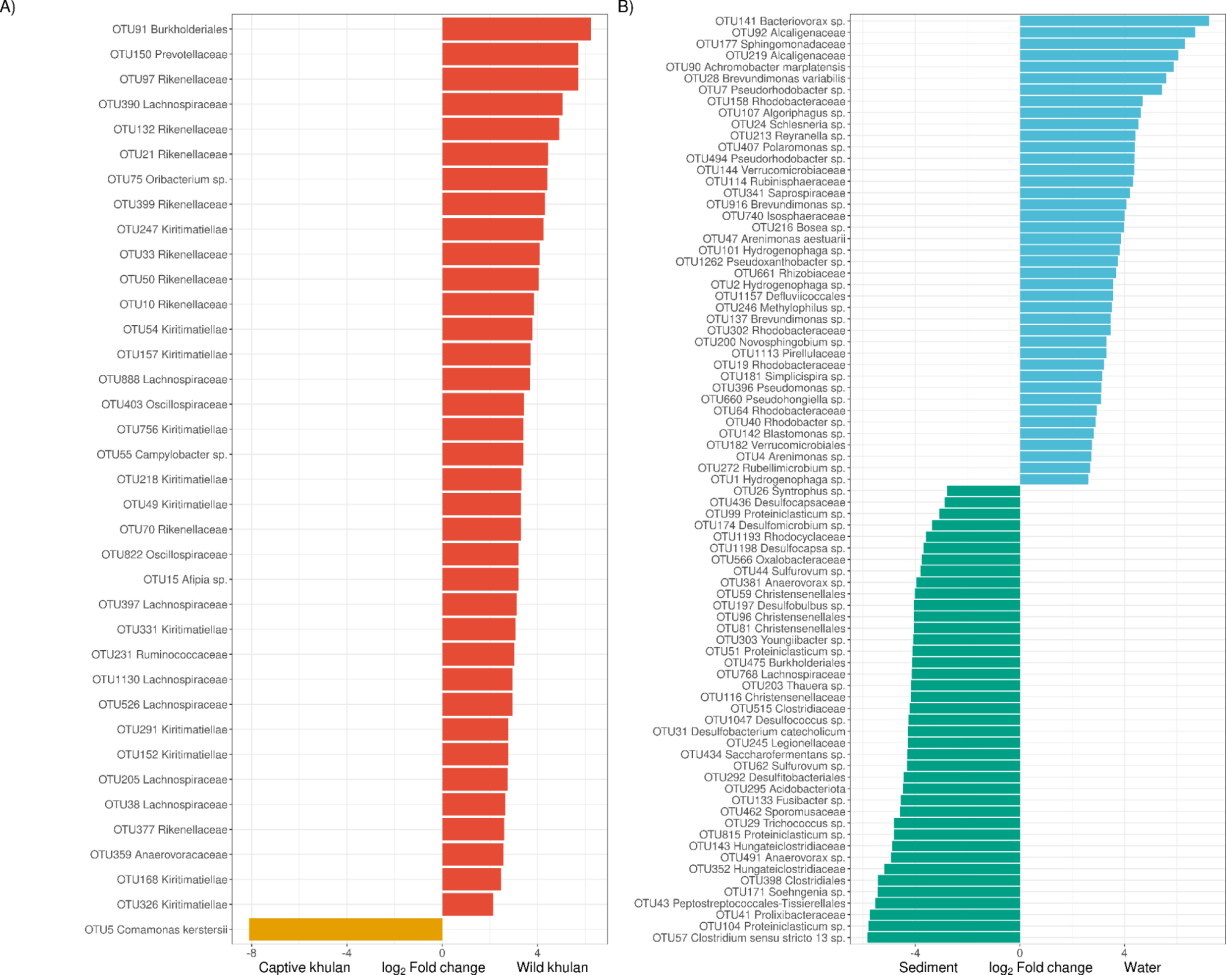
Detection of differentially abundant bacteria between sample groups. (**A**) Among shared OTUs between wild and captive khulans, OTUs belonging to the families Lachnospiraceae, Rikenellaceae and class Kiritimatiellae are significantly more abundant in wild khulan microbiomes, while just *Comamonas kerstersii* is abundant in captive populations. (**B**) Water and sediment microbiomes show characteristic bacteria each, while water has significantly more abundant *Bacteriovorax* sp. and other Proteobacteria OTUs, members of the phyla Firmicutes from the family Clostridiaceae, are more abundant in sediment.

The goal was to identify bacterial groups characterizing the microbial communities of waterholes and khulans and to assess whether potentially pathogenic bacteria were present. We conducted a differential abundance analysis to achieve this, comparing wild-captive and water-sediment samples. Of the 726 shared OTUs between wild and captive khulans, 37 were significantly enriched in one or the other group. Notably, most of these OTUs (36 out of 37) were enriched in wild khulan microbiomes but depleted in microbiomes from captive khulans (Fig. 3A). For instance, the order Burkholderiales was 75 times more abundant in wild khulan microbiomes than captives (Log2FC = 6.239, W = 7.562, p.adj = 1.018E-11). Burkholderiales also had a higher prevalence in wild khulans (21 out of 21, 100%) than captive khulans (4 out of 12, 33.3%). Furthermore, several other bacterial groups represented around 70% of the significantly enriched bacteria in wild khulan microbiomes but were depleted in captive khulan microbiomes. These included eleven OTUs from the class Kiritimaiellae, eight OTUs from the family Rikenellaceae, and seven OTUs from the family Lachnospiraceae. On the other hand, *Comamonas kerstersii* was found to be 276 times more abundant in captive khulans than in wild ones (Log2FC= -8.11, W= -6.654, p.adj = 2.921E-11). Its prevalence in microbiomes from captive khulans (10 out of 12, 83.4%) was also significantly higher than in wild kuhlan microbiomes (10 out of 21, 47.6%). These findings highlight distinct differences in microbial composition between wild and captive khulans, with certain bacterial groups showing significant enrichment in either population. This information contributes to our understanding of the potential impact of captivity on khulan microbiomes and can aid in managing and conserving these unique animals.

A clear differentiation between water and waterhole sediment microbiomes was represented primarily by the phylum Proteobacteria, specifically Firmicutes (Figure S4). In water, members from the families Rhodobacteraceae (8 OTUs) and Comamonadaceae (5 OTUs) accounted for more than 30% of the differentially abundant OTUs in that sample type. *Bacteriovorax* sp. was not only the most differentially abundant bacterium in water, with levels up to 150 times greater than in sediment (Log_2_FC = 7.234, *W* = 6.850, *p.adj* = 7.411E^− 12^), it was also more prevalent in water (18/25, 72%) compared to sediment (7/25, 28%). Out of the 41 OTUs differentially abundant in water, only three were classified at species level: *Achromobacter marplatensis*, *Brevundimonas variabilis* and *Arenimonas aestuarii*; none of those were recognized as waterborne pathogens but rather as aerobic, mesophilic environmental bacteria (Fig. 3B). OTUs from the families Christensenellaceae (4 OTUs) and Clostridiaceae (7 OTUs) were more abundant in sediment than in water microbiomes. The most differentially abundant OTU in sediment corresponded to *Clostridium sensu stricto* 13 (Log_2_FC= -5.843, *W*= -6.276, *p.adj* = 1.793E^− 8^) being 57 times more common in sediment than in water. The mesophilic anaerobe *Desulfobacterium catecholicum* was the only bacterium identified at the species level. It was 19 times more abundant in sediment than in water (Log_2_FC= -4.283, *W*= -5.383, *p.adj* = 1.571E^− 8^) and has a prevalence of up to 92% (23/25) in sediment samples. *Hydrogenophaga* sp. (OTU1), a family Comamonadaceae member, despite being more abundant in water microbiomes (Log_2_FC = 2.63, *W* = 5.228, *p.adj* = 3.188E^− 6^); was the only OTU present in both water and sediment.

### Microbial composition in wild khulans is more similar to that of captive khulans than to domestic or wild horses from the same region

**Fig. 4:**
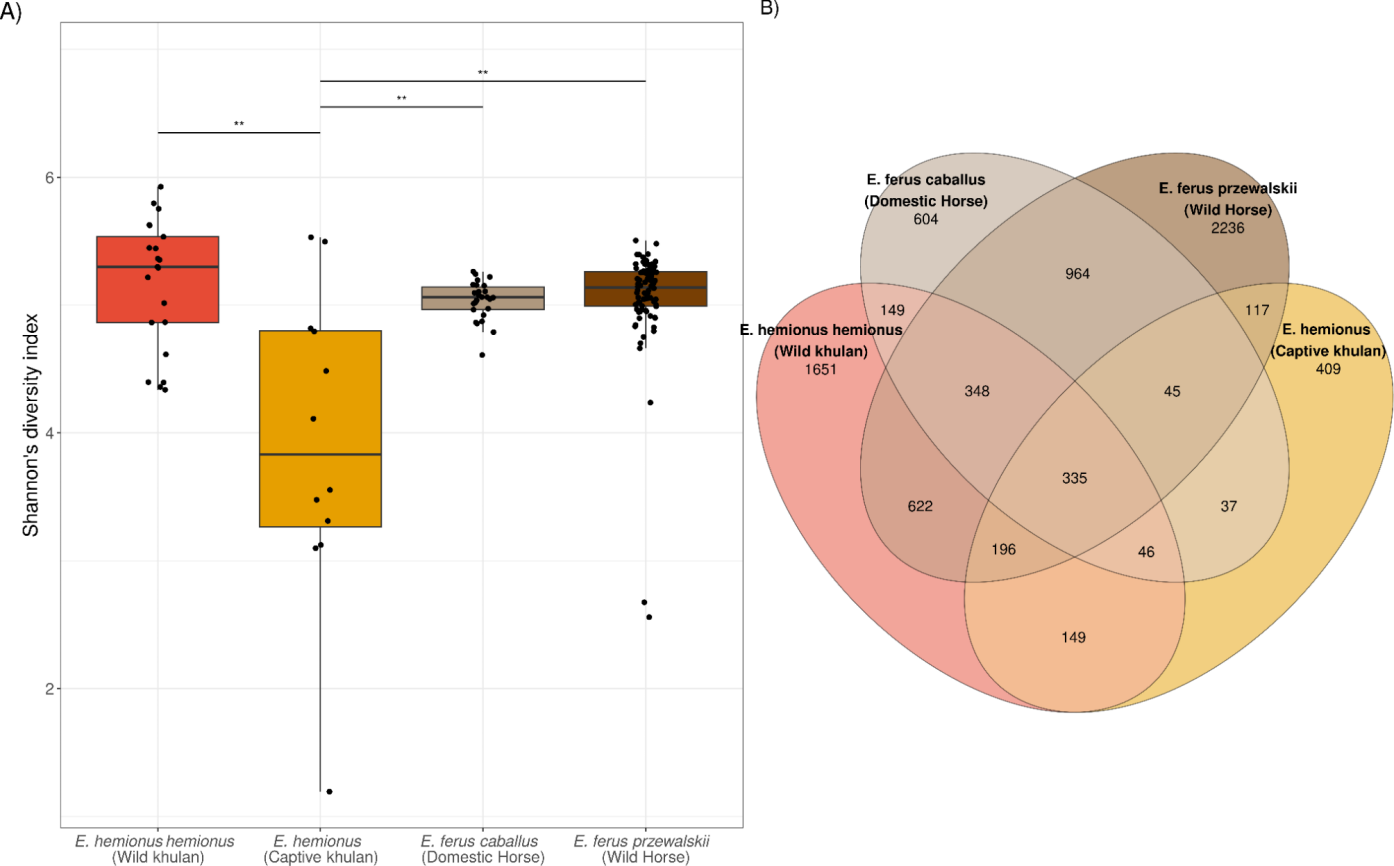
Alpha diversity metrics between microbiomes from wild, captive khulans and wild, domestic horses. (**A**) Captive khulan microbiomes are significantly less diverse than those from wild populations of khulans and wild and domestic horses from Mongolia^7^. (**B**) Venn diagram showing the overlap in OTUs among wild khulans, wild horses, captive khulans, and domestic horses. Wild khulans and wild horses show the highest number of unique OTUs compared to either captive khulans or domestic horses. Wild khulans have 1,651 unique OTUs and wild horses have 2,236, while both domestic horses and captive khulans have fewer than 700 unique OTUs each. A significant overlap of 335 OTUs might indicate a core set of bacteria shared among Mongolian equids.

To test whether wild khulan microbiomes in our study were more similar to other wild equid populations from the same region, we compared our data against domestic and Przewalski’s horse microbiomes from an external study^7^. Shannon’s diversity index comparisons indicated that captive khulan microbiomes are significantly less diverse than those of wild khulans but also than those of horses, both domestic (Mann-Whitney *U*,* U*(*n*_captive khulans_= 12, *n*_domestic horses_= 26) = 56, *r =* 0.509, *p*_*FDR.adjs*_ = 0.003) and wild (Mann-Whitney *U*,* U*(*n*_captive khulans_= 12, *n*_wild horses_= 82) = 191, *r =* 0.352, *p*_*FDR.adjs*_ = 0.003) (Fig. 4A). Conversely, the Chao1 index showed no significant difference in richness between captive khulan microbiomes and those of domestic (Mann-Whitney *U*,* U*(*n*_captive khulans_= 12, *n*_domestic horses_= 26) = 100, *r =* 0.285, *p*_*FDR.adjs*_ = 0.103) and wild (Mann-Whitney *U*,* U*(*n*_captive khulans_= 12, *wild*_horses_= 82) = 340, *r =* 0.178, *p*_*FDR.adjs*_ = 0.103) horse microbiomes. Wild and captive khulan shared 335 OTUs with Przewalski’s and domestic horses and this overlap is greater than predicted by chance (Fisher’s exact test, DF = 12220, Odds ratio = 6.399, Overlapping *p* = 1.3e^− 106^), suggesting that a core set, potentially keystone species, of bacteria can be identified in Asian equids (Fig. 4B). Fourteen OTUs reached taxonomic annotation at the species level: *Bacteroides fluxus*, *B. uniformis* and *B. caccae* from the family Bacteroidaceae, *Intestinimonas butyriciproducens* and *Flavonifractor plautii* from the family Oscillospiraceae, *Comamonas kerstersii* and *Caenimonas terrae* from the family Comamonadaceae, in addition to *Clostridium sartagoforme*,* Erwinia billingiae*,* Ralstonia insidiosa*,* Enterocloster* [*Clostridium*] *aldenense*, *Pasteurella aerogenes*,* Escherichia coli* and *Streptococcus equinus*, all of them mesophilic bacteria known from mammalian hosts or the environment.

**Fig. 5:**
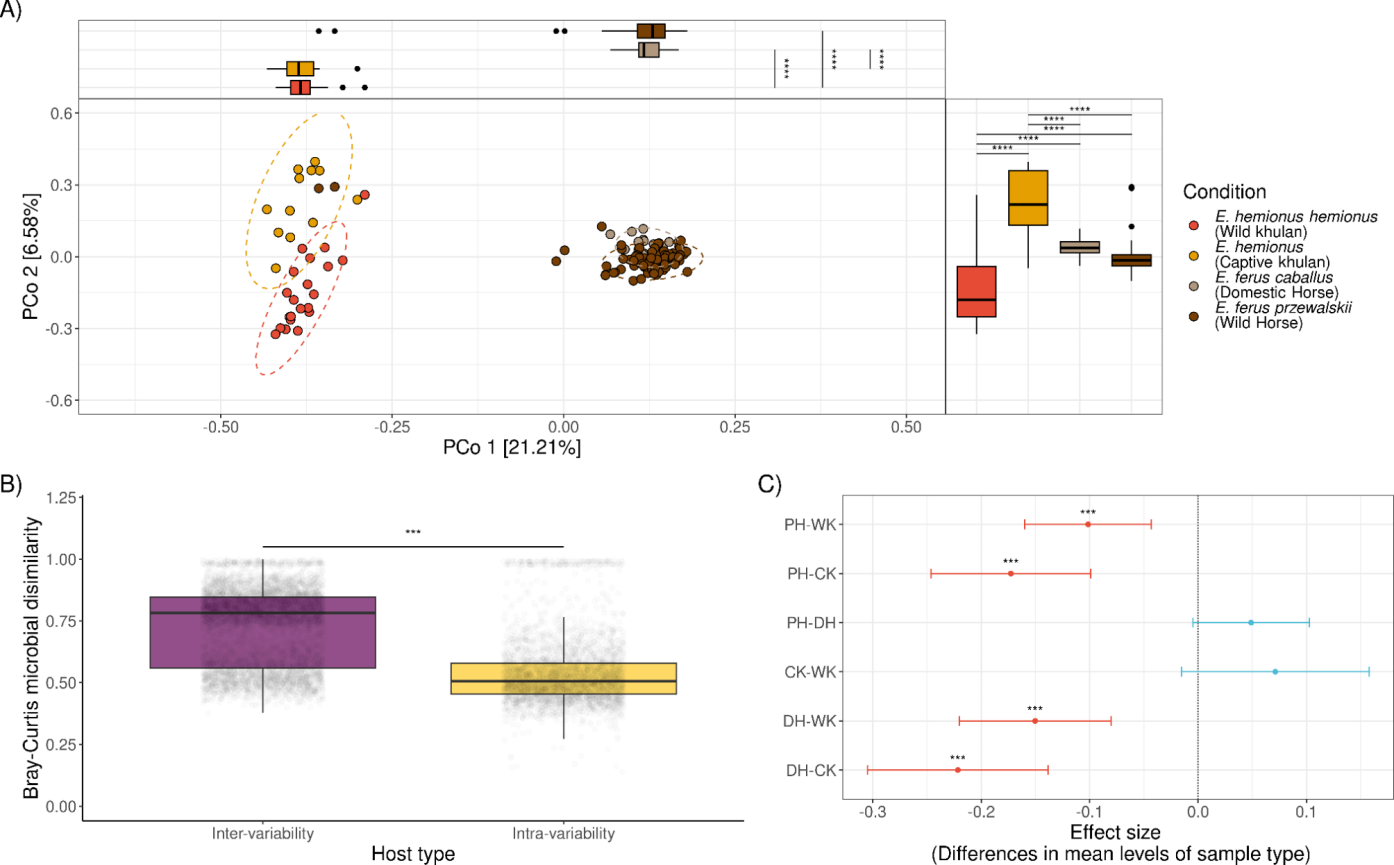
Beta diversity analysis shows distinct microbial communities from wild and captive khulans compared to domestic and wild horses from a near region. (**A**) Principal Coordinates Analysis (PCoA) plots comparing the microbial beta diversity of wild and captive khulans, domestic and wild horses from Metcalf et al., 2017. Both sample populations clustered separately, confirming distinctive compositions between studies. Clustering of khulan and horse microbiomes in the PCoA significantly depends on sample type (condition), either wild, captive kulans or wild or domestic horses. Significance codes: ‘****’ 0.0001, ‘***’ 0.001 ‘**’ 0.01 ‘*’ 0.05 (**B**) Pairwise comparison of intersample Bray-Curtis (BC) dissimilarity distances shows strong differences within each khulan or horse’s microbial communities. Every dot represents the distance between a pair of samples. Inter host type variability reflects larger microbial dissimilarity between host types than within host types. (**C**) Mean and the 95% confidence interval of a post-hoc Tukey’s HSD test performed on BC dissimilarity distances shows highly significant differences between wild khulans (WK) and captive khulan (CK) microbiomes to domestic (DH, E. *caballus*) and Przewalski’s horses (PH, *E. ferus przewalskii*) microbiomes.

PCoA based on the BC distance was used to determine how similar the bacterial composition of khulans and horses was. PCoA showed three separate clusters, representing the two khulan-derived microbiomes, either wild or captive, and an additional cluster with overlapping wild and domestic horse microbiomes (Fig. 5A). PERMANOVA indicates that the sample type (living condition), wild khulan, captive khulan, domestic horse, or Przewalski’s horse, was a significant predictor of differences in microbial composition, explaining around 21.5% of the total variation (Table 4).

GLMMs including intra-variability or inter-variability within host type (wild Khulan, captive khulan, wild horse, domestic horses) or study of origin (own and Metcalf et al., 2017) as predictors of microbiome dissimilarity were used to determine which of these characteristics was more influential in the microbiome composition differences. The microbial dissimilarity was significantly greater between host types than within host types and this factor explains 9.3% of variation (LRT: *χ*^2^ = 756.2, df = 1, *p* < 2.2e-16, *r*^*2*^ = 0.0932). This is reflected by the median BC distance above 0.75 and closer to 1.0 for the inter-host type comparisons (Fig. 5B). Study of origin (LRT: *χ*^2^ = 8.1226, df = 1, *p* = 0.004, *r*^*2*^ = 0.0015) was also significant, but did not explain more than 1% of the total variation.

Pairwise multiple comparison of mean differences between sample types revealed a significant distinction between the microbiomes of free-living khulans and both domestic and free-living horses (Tukey HSD, Mean diff_wild khulan−Domestic horse_= -0.15, -0.08 - -0.22–95% CI, *p.adj* = 7.3e-7; Mean diff_wild khulan−wild horse_= -0.101, -0.043 - -0.159 95% CI, *p.adj* = 7.8e-5). Microbiomes from wild and captive khulans and horses did not significantly differ, suggesting the closer microbiome relationship among species than between species (Mean diff_wild horse−Domestic horse_= 0.049, -0.005–0.103 95% CI, *p.adj* = 0.088) (Fig. 5C). Despite a core set of shared OTUs among equids, wild and captive khulan microbiomes are distinct from domestic horses (*E. caballus*) or Przewalski’s horses.

**Table 4:** PERMANOVA based on the Bray-Curtis distances between wild and captive khulans and wild and domestic horses from Metcalf et al., 2017.

	Df	Sums Of Sqs	F-Model	*R* ^2^	*p*
Sex	1	0.202	1.187	0.0066	0.19
Living condition	2	7.295	21.475	**0.228**	0.001***
Residuals	136	23.098	-	0.722	-
Total	140	32.012	-	1.0000	-

---.

Significance codes: ‘***’ 0.001, ‘**’ 0.01, ‘*’ 0.05, ‘.’ 0.1, Df: Degrees of freedom, *F*-Model: *pseudo F*-test statistic, R^2^: Variance explained and corresponding *p* value based on 999 permutations.

## Conclusion

We demonstrate limited microbial sharing between khulans and waterholes they drink from, challenging the hypothesis that these act as a source of their gut bacteria or that they shape the water microbiome through their exudates. Furthermore, we observe that captive khulans exhibit lower microbial diversity compared to their wild counterparts. However, captive and wild khulans are more similar to each other than to domestic horses and Przewalski’s horses with whom they share parts of their range in Eurasia demonstrating that species specific microbiome adaptation is a more important determinant of microbial community structure than captivity. These findings highlight the complex interplay between host genetics, diet, environmental factors, and captivity in shaping the khulan microbiome. Further research is needed to elucidate the underlying mechanisms driving microbial variation and its implications for khulan health and conservation.

## Discussion

Long-term co-evolutionary interaction between hosts and microbes shapes microbial diversity and structure of mammalian microbiomes. While host genetics, domestication processes, and the environment influence the symbiotic host-microbiome interaction, the extent of microbial sharing between hosts and the environment remains unclear^11,12^. In this study, we investigate the contribution of environmental microbiota from freshwater bodies in the Mongolian Gobi to the gastrointestinal microbiomes of khulans. We also explore the impact of captivity far from the habitat of origin and domestication on microbiomes by comparing them with those of captive khulans in Europe and two other Asian equid species in Mongolia.

One expectation regarding microbiome-host sharing is that environmental sources, including water, can act as reservoirs of microbes, some of which may potentially be pathogenic. However, our results show minimal sharing of microbial taxa between khulan microbiomes and the environmental samples. This finding is surprising, as one might expect the water to exhibit a fecal microbiome profile due to khulan waste which accumulates near and sometimes in the water. In contrast, wastewater from human settlements often shows a microbial profile similar to human feces^13^. These observations may suggest that khulans avoid excessively contaminating their water sources. This will need to be confirmed by future khulan behavioral studies.

Previous analyses of wild equids’ microbiomes have shown that differences depend highly on the habitat or geographical region of origin. For Przewalski horses, the food available in the region determines their microbiome^14^. In our study, the geographical region did not significantly predict gut microbial differences in khulan. These equids can move over hundreds of kilometers, making it challenging to define individual diets; although population level diets are different in the two study areas in the Gobi^15^. Ang et al.^16^ observed a clear distinction in the microbiome composition of wild equid populations from Japan, Argentina and Europe. Thus, large geographical distances at the country or continental level determine microbiome distinction derived from different food sources, which are likely not observed at the regional or home-range distribution level.

Khulans are hindgut fermenters able to digest complex carbohydrates like cellulose. Ruminococcaceae and Fibrobacteraceae, although low in abundance in equid microbiomes, are crucial for hindgut plant-wall degradation^17–20^. Our study identified specific OTUs assigned to Ruminococcaceae and Fibrobacteraceae in wild and captive khulans, with varying prevalence and abundance. Notably, these OTUs were not detected or were rare in the environmental samples, highlighting their specificity to equine microbiomes and limited persistence in water bodies. Furthermore, wild khulans exhibited enrichment of Firmicutes and Bacteroidota, particularly Lachnospiraceae and Rikenellaceae, which present the ability to metabolize complex carbohydrates and produce metabolites like short-chain fatty acids (SCFAs)^21,22^. This enrichment in SCFA producers could be an adaptation to the stressful nutritional conditions in the Gobi desert, similar to observations in wild camels^23^. These findings suggest that the khulan microbiome exhibits specific adaptations in response to the challenging nutritional environment, contributing to their ability to thrive in the arid regions of Mongolia.

Captive animals generally exhibit lower microbial diversity than their wild counterparts, which aligns with our findings for khulans. We observed a significantly lower richness and microbial diversity in captive animals. Our results show that around 20% of the microbial composition changes were due to wild versus captive living conditions for khulans (19.4%) and horses (20.3%). This decrease in richness and diversity in captive animals is likely due to dietary changes. The diet in captivity, often rich in starch and glycogen from cereal and grains, differs significantly from the natural diet of wild equids, primarily consisting of grass cellulose (Bulmer et al., 2019). These dietary variations can profoundly affect the microbiome’s composition and function. The lack of microbial functions like SCFA production or enrichment of antimicrobial resistance genes is another factor associated with dysbiosis of the microbiome in captive horses^20,24^.

Contrary to the expectation that captivity shapes microbiomes similarly, our study reveals that wild khulans are more similar to captive khulans than wild horses to captive horses^7^. Only a small percentage of shared OTUs were observed among the three equid species (khulan, domestic horses, and Przewalski’s horses), which is unexpected due to their close phylogenetic and ecological relationship and shared digestion physiology. These findings suggest that while captivity does influence the khulan microbiome, other factors such as evolutionary history, ecological niche, or host-specific traits may outweigh captivity.

The finding of a lack of significant difference between wild and captive khulan microbiomes, suggesting the closeness of both microbiomes, may have several implications related to keystone species in the microbiome and genetically determined species-specific components^25^.

In the case of khulans, the closeness observed between wild and captive microbiomes may indicate the retention of keystone species despite the differences in their respective environments. It suggests that certain microbial species within the khulan gut might be essential for the animal’s health and survival, regardless of whether they are in their natural habitat or captivity. The presence and stability of these keystone species may be crucial for the khulan’s ability to adapt to varying nutritional conditions and maintain its health^26^.

## Materials and methods

The experimental procedure involves extraction and amplification of the 16S rRNA gene from bacterial DNA obtained from rectal swabs of wild equids, as well as water and sediment samples collected in Mongolia (specifically Gobi A and Gobi B), followed by PacBio sequencing using the Sequel System.

### Sample collection

Samples were collected from Mongolia in two different areas: (1) The Southern Gobi (42.9400°N, 108.4673°E) in October 2015 and (2) The Great Gobi B Strictly Protected Area (45.1882°N, 93.4288°E) between June – July 2016.

### Khulan samples

Twenty-one wild khulans, were sampled in Southern Gobi, Mongolia, in October 2015. Animals were anesthetized for GPS-satellite collaring^27^ which also allowed to collect rectal swabs. Rectal swabbing was performed using sterile UTMTM Viral Transport Media swabs (Copan Diagnostics Inc., Murrieta, California, USA). Samples were stored at -20 °C in Mongolia, subsequently transported to the Research Institute of Wildlife Ecology, University of Veterinary Medicine, Vienna, and stored at − 80 °C.

The same collection procedure was performed to obtain samples from 12 captive khulans in the Serengeti Park Hodenhagen, Germany, in 2017.

This study was conducted in accordance with the ARRIVE guidelines. Ethical review and approval were granted by the ethic commission at the Leibniz Institute for Zoo and Wildlife Research, approval no: 2017-02-03. Additionally, the ethic commission at the University of Veterinary Medicine Vienna was informed and provided general consent (ETK-15/03/2016). All captures and animal handling procedures were performed in strict accordance with relevant guidelines and regulations. Capture activities were authorized by the Mongolian Ministry of Nature, Environment, and Tourism under capture permits 6/4136 issued on 2013/08/01, and 5/5656 issued on 2015/09/17.

### Water and sediment

In the Great Gobi B ten water and sediment samples have been collected, while 14 water and sediment samples were collected in South East Gobi. 50 ml of water was filtered through a 0.2 μm Sterivex filter unit (Millipore, USA) using disposable syringes. For sediment collection, 25 g from the first 1–3 cm of sediment at the bottom of the water holes was collected. Following collection, samples were stored on ice and frozen at -20 °C within six hours.

Samples were transported to the Leibniz Institute for Zoo and Wildlife Research (IZW), Germany, where they were stored until further laboratory analysis, therefore keeping the cold chain in place from Mongolia to Germany.

### DNA Isolation

#### Khulan samples

DNA from rectal swabs was extracted using the QIAamp DNA Stool Mini Kit (QIAGEN, Hilden, Germany) according to the manufacturer’s instructions.

#### Water and sediment

Microbiomes from water were obtained using a 0.2 μm Sterivex filter unit (Millipore, USA) that was cut into pieces with a sterile scalpel blade. The resulting filter pieces were placed into 1.5 ml microfuge tubes with 5 µg of low-binding zirconium glass beads (OPS Diagnostics, NJ, USA). The QIAamp DNA Mini Kit (Qiagen, Germany) was then used to extract DNA with the following modifications: 350 µl ATL lysis buffer plus 50 µl of proteinase K were added in the initial lysis and vortexed at 3,000 rpm for 5 min at 56 °C with an Eppendorf MixMate^®^ 175 (Eppendorf, Hamburg, Germany). Following centrifugation, the DNA was eluted in 100 µl. The DNA from sediment was extracted using the Machery-Nagel NucleoSpin^®^ Soil kit. The recommended protocol for the kit was followed using 500 mg of sediment. The DNA concentration was determined using the Agilent Tapestation with Genomic tapes and reagents (Agilent Technologies, USA).

### Amplification and sample tagging of the 16S rRNA gene

Specific primers, originally designed by Wagner et al. 2016^28^, were used to amplify the full 16S rRNA gene (~ 1,500 bp). Each forward and reverse primer was tagged with a specific 16 bp sequence to identify samples when pooled for PacBio sequencing (Table S1). The PCR reactions were run in triplicate, with a total reaction volume of 20 µl per sample. Each reaction contained the following per sample: 0.75 µl of each forward primer 5`-AG RGT TYG ATY MTG GCT CAG-3` and reverse primer 5`-RG YTA CCT TGT TAC GAC TT-3`, 0.7 µl BSA, 12.5 µl of 2X MyFi mix (Bioline, Germany) and 5.3 µl of nuclease-free water. The following thermal cycler conditions were used to amplify a ~ 1,500 bp product: 95 °C for 3 min, followed by 25 cycles denaturation 95 °C for 30 s, annealing 57 °C for 30 s, and extension 72 °C for 60 s and a final extension at 72 °C for 3 min. The triplicate PCR products were then pooled. Eight equally pooled samples were purified using 20 µl AMPure XP beads (Agencourt Bioscience) according to Illumina’s 16S rRNA metagenomic sequencing library preparation protocol. The DNA concentration was determined with the Agilent Tapestation using the D5000 tapes and reagents (Agilent Technologies, USA).

### PacBio 16S rRNA amplicon sequencing

PCR products from each of the three sample types (sediment, water and swabs) were pooled together, resulting in three pooled samples. The three pools were processed by the Max Delbrück Center, Berlin, for PacBio library construction and sequencing. Pools were individually purified using AMPure XP beads (Beckman Coulter) at a concentration of 0.9X. Libraries were then created for each sample pool using the PacBio (Pacific Biosciences, Menlo Park, CA) 5 kb template preparation protocol and the SMRTbell™ Template Prep Kit 1.0, following the manufacturer’s guidelines. The length and concentration of the libraries were then determined with the 2100 Agilent Bioanalyzer using the 1200 DNA chemistry (Agilent Technologies). Sequencing on the PacBio Sequel system was performed using the MagBead Standard protocol, C4 chemistry and P6 polymerase on a single v3 Single-Molecule Real-Time (SMRT) cell with 1 × 180 min movie for each sample. Each individual pool was run on a SMRT cell.

### Sequence analysis

The reads from the insert sequence were processed within the SMRT^®^ Portal browser (minimum full pass = 1; and a minimum predicted accuracy of 90).

The circular consensus sequences generated for this study have been deposited in the National Center for Biotechnology Information’s (NCBI) Sequence Read Archive (SRA) database under the Bioproject accession number PRJNA1002227.

The amplicon sequences are filtered for human (GRCh38.p13) and horse (EquCab3.0) sequences as the closest host reference genome available, using bbtools v. 38.87^29^. Filtered reads were analyzed using LotuS pipeline v. 2.16^30^. Quality filtering is performed using SDM with LotuS defaults for PacBio sequences. Operational taxonomic units (OTUs) were clustered using cd-hit. OTU annotation is performed using lamba with SILVA database v. 138^31^.

### 16S rRNA sequencing data curation

All OTUs and their taxonomic annotation were compiled with the sample metadata into a single object for further analysis using the package *Phyloseq* v1.34.0^32^. After a further curation process was performed, the final dataset was generated: OTUs with zero counts or singletons were removed. A taxonomic filter was based on discarding non-bacterial or unassigned OTUs at the phylum level. A supervised prevalence filtering removed low-prevalent OTUs in less than five samples. This dataset was rarefied to the minimum library size and used for further alpha diversity estimation. Finally, OTUs in the rarefied dataset were subsetted from the raw counts and then normalized by transforming OTUs proportions by sample to an even depth (1E6) for beta diversity estimations.

### Statistical analysis

All downstream statistical analyses were performed in R [v4.0.3]^33^. Richness (Chao1 index) and diversity (Shannon Index) were used to estimate alpha diversity metrics for each sample. Richness and diversity were calculated on the rarefied dataset using *Microbiome* v1.17.2^34^. Beta diversity analysis was performed to compare the microbial communities of samples with different origins (wild and captive khulans, and water or sediment from waterholes). Pairwise comparisons of alpha diversity metrics within sample types were performed using the Mann-Whitney *U* test and Benjamini-Hochberg correction^35^ to adjust for multiple comparisons, using the R package *rstatix* v0.7.0^36^. Correlations between library size (read counts per sample) and alpha diversity measurements were estimated using Spearman’s correlation tests implemented using the *stat_cor* function within the R package *ggpubr* v0.4.0^37^. Bray–Curtis (BC) dissimilarity distances were calculated as a metric to assess differences in microbial composition and visualized using principal coordinate analysis (PCoA) in the package *Phyloseq* v1.34.0^32^. Permutational multivariate analysis of variance (PERMANOVA) was used to compare BC dissimilarity indices and test multivariate marginal effect of sample types in general and sample-specific associated metadata with the function *adonis2*. A total of 999 permutations and stratification for sample origin (khulan or waterhole) were applied for the analysis. Analysis of multivariate homogeneity of sample type dispersion was performed using BC dissimilarity distances with *vegan* v2.5-7^38^. Confidence intervals on the differences between the mean distance-to-centroid of the different sample types were estimated and statistical differences among sample types were tested using Tukey’s ‘Honest Significant Difference’ method. General linear mixed-effects models (GLMMs) were fitted to test whether wild or captive khulan microbiomes were more similar to water or sediment microbiomes using ranked BC dissimilarities as response, living condition (wild or captive) as predictor (fixed effect) and samples as random effect to control for pseudoreplication. Statistical models were fitted using the package *lme4* v1.1-28^39^. Likelihood ratio test (LRT) against the null model was used to determine the significance of the fixed effect. LRT was performed using package lmtest v0.9-39^40^. Differential abundance analysis was performed using the *DESeq2* package^41^ employing OTU raw counts of shared OTUs with the following group comparisons: wild - captive khulan microbiomes and water - sediment waterhole. Contrast comparison was done based on Wald test. The *p*-values are corrected for multiple testing using the FDR method. Differentially abundant OTU were considered significant at adjusted *p*-value < 0.001 and with log_2_ fold change higher to 0.6 or lower to -0.6.

Geographical distance khulan-waterholes were estimated using the package *geosphere* v1.5-14^42^. We considered the coordinates from the centroid of the area covered by each khulan to the waterholes. The potential of visits from each khulan to a waterhole was estimated based on their geographical distance. Waterholes in the range from the South Gobi were considered “potential visits”, while those from the Great Gobi B were considered as non-visit. Comparison of microbial composition and geographical distances between wild khulans and waterhole samples were computed using Mantel test with 999 permutations implemented in *vegan* v2.5-7^38^.

To compare khulan microbiomes to an external equine dataset, the data from Metcalf et al., 2017 was processed with the same conditions as for the dataset from this study. Alpha and beta diversity metrics were estimated as described above. GLMMs were fitted to test whether microbiomes from the same host type were more similar among them than between them using ranked BC dissimilarities as response, host type (wild khulan, captive khulan, domestic horse, wild horse), and study of origin (own and Metcalf et al., 2017) as predictors (fixed effect), as before sample ID was used as random effect to control for pseudoreplication. Fisher Exact Test was implemented to estimate the significance of OTUs overlap between khulan and horses datasets with the function *testGeneOverlap* in the GeneOverlap package v1.32.0^43^.

All plots were created using the R package *ggplot2* v3.3.5^44^.

## Electronic supplementary material

Below is the link to the electronic supplementary material.

Supplementary Material 1

Supplementary Material 2

Supplementary Material 3

Supplementary Material 4

Supplementary Material 5

Supplementary Material 6

Supplementary Material 7

## Data Availability

The circular consensus sequences generated for this study have been deposited in the National Center for Biotechnology Information’s (NCBI) Sequence Read Archive (SRA) database under the Bioproject accession number PRJNA1002227.

## Abbreviations

BC: Bray–Curtis (dissimilarity)
FDR: false discovery rate
GLMMs: General linear mixed-effects models
LRT: Likelihood ratio test
OTU: Operational taxonomic unit
PCoA: principal coordinate analysis
PERMANOVA: Permutational multivariate analysis of variance
SMRT: Single-Molecule Real-Time
