# Supplementary figures and images for: Unraveling the distinctive gut microbiome of khulans (Equus hemionus hemionus) in comparison to their drinking water and closely related equids
Source: Sci Rep. 2025 Jan 22;15:2767. doi: 10.1038/s41598-025-87216-z (PMC11754619; doi:10.1038/s41598-025-87216-z)

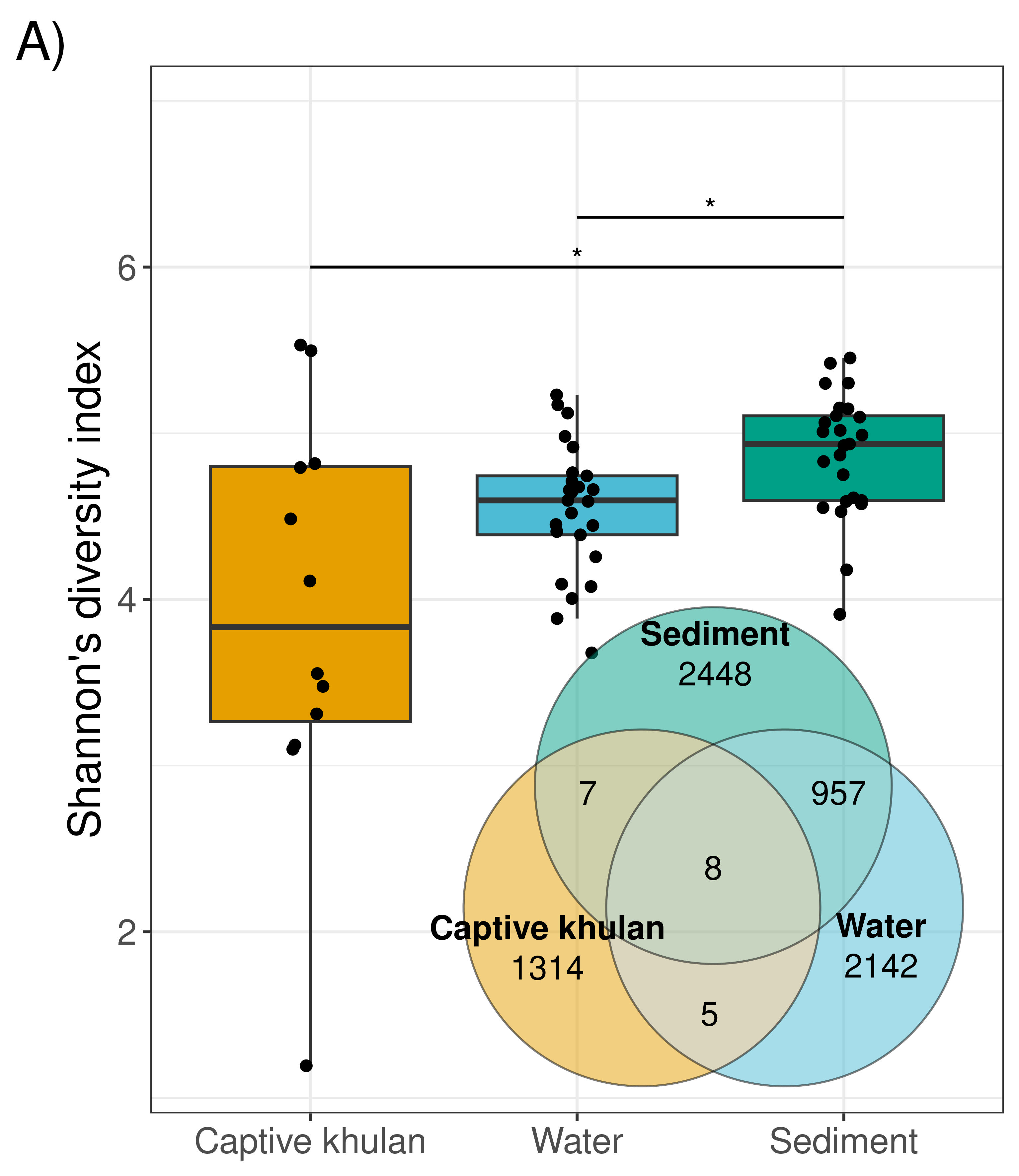

Supplement: Supplementary file 2 — Supplementary Material 2 [file 41598_2025_87216_MOESM2_ESM.tiff]
